# Planning to be routine: habit as a mediator of the planning-behaviour relationship in healthcare professionals

**DOI:** 10.1186/s13012-017-0551-6

**Published:** 2017-02-21

**Authors:** Sebastian Potthoff, Justin Presseau, Falko F. Sniehotta, Marie Johnston, Marko Elovainio, Leah Avery

**Affiliations:** 1 Institute of Health and Society, Faculty of Medical Sciences, Newcastle University, Newcastle upon Tyne, NE2 4AX UK; 2 Clinical Epidemiology Program, Ottawa Hospital Research Institute, Ottawa, Canada; 3 School of Epidemiology, Public Health and Preventive Medicine, University of Ottawa, Ottawa, Canada; 4 Institute of Applied Health Sciences, College of Life Sciences and Medicine, University of Aberdeen, Aberdeen, UK; 5 University of Helsinki, Helsinki, Finland; 6 Institute of Cellular Medicine, Newcastle University, Newcastle upon Tyne, NE2 4HH UK

**Keywords:** Habit, Intention, Action planning, Coping planning, Healthcare professionals, Implementation intentions, Automaticity, Primary care, Type 2 diabetes

## Abstract

**Background:**

Gaps in the quality of care provided to people with type 2 diabetes are regularly identified. Healthcare professionals often have a strong intention to follow practice guidelines during consultations with people with type 2 diabetes; however, this intention does not always translate into action. Action planning (planning when, where and how to act) and coping planning (planning how to overcome pre-identified barriers) have been hypothesised to help with the enactment of intentions by creating mental cue-response links that promote habit formation. This study aimed to investigate whether habit helps to better understand how action and coping planning relate to clinical behaviour in the context of type 2 diabetes care.

**Methods:**

The study utilised a prospective correlational design with six nested sub-studies. General practitioners and practice nurses (*n* = 427 from 99 UK primary care practices) completed measures of action planning, coping planning and habit at baseline and then self-reported their enactment of guideline-recommended advising, prescribing and examining behaviours 12 months later. Bootstrapped mediation analyses were used to test the indirect effect of action and coping planning on healthcare professionals’ clinical behaviour via their relationship with habit.

**Results:**

Healthcare professionals who reported higher degrees of action or coping planning for performing six guideline recommended behaviours in the context of type 2 diabetes care were more likely to report performing these behaviours in clinical practice. All 12 bootstrapped mediation analyses showed that the positive relationship between planning (action and coping planning) and healthcare professionals’ clinical behaviour operated indirectly through habit.

**Conclusions:**

These findings suggest that habit mediates the relationship between planning (action and coping planning) and healthcare professional behaviour. Promoting careful action and coping planning may support routinised uptake of guideline-recommended care by healthcare professionals in the primary care setting. Given the competing demands on healthcare professionals, exploring the behavioural processes involved in promoting more routinisation of behaviours where possible and appropriate could free up cognitive capacity for clinical behaviours that rely on more deliberation.

**Electronic supplementary material:**

The online version of this article (doi:10.1186/s13012-017-0551-6) contains supplementary material, which is available to authorized users.

## Background

Type 2 diabetes is a worldwide health issue affecting approximately 415 million people between the ages of 20 and 70 years in 2015 [1, 2]. In the UK alone, the number of diagnosed cases has doubled from 1.4 million in 1996 to 3.5 million in 2015 [1]. Whilst poor management of type 2 diabetes can lead to serious complications such as cardiovascular disease [3, 4], there is considerable evidence that successful management can decelerate, halt progression and, in some cases, even reverse the condition through health behaviour change [5]. Although there are national clinical practice guidelines for type 2 diabetes (e.g. UK [6], USA [7], Canada [8] and Australia [9]), the implementation of these guidelines into clinical practice is frequently suboptimal [10]. For example, a national diabetes audit in the UK showed that only 59% of patients received all eight guideline recommended care processes (e.g. blood test for glucose control and foot examination for foot ulcer risk) [4].

Well-tested theories from behavioural science can inform implementation interventions to modify healthcare professionals’ behaviours and explore mediating mechanisms and potential moderators of such interventions [11–13]. Predominant theories of behaviour used in implementation science tend to propose that healthcare professional behaviour is determined by a reflective process of active decision-making [14]. Other approaches (i.e. dual process models) acknowledge that behaviour is driven by more than one system [15–19]. According to these models, there are two systems of mental processing: a reflective system that is slow and effortful and is mainly engaged in conscious rational decision-making and an impulsive system that operates quickly and efficiently on a non-conscious level [17]. This dual processing approach can be useful for informing implementation research, and interventions may be well-served to focus not only on changing the reflective pathway by educating and motivating healthcare professionals but also on the role of impulsive processes [20, 21].

One variable that represents the impulsive pathway to behaviour is habit. Healthcare professionals often perform the same clinical behaviours repeatedly until they become routine practice, and once a behaviour has become routine, it is increasingly controlled by habit rather than solely by conscious, in the moment decision-making. From a psychological perspective, habit can be defined as *‘a process by which a stimulus automatically generates an impulse towards action, based on learned stimulus-response associations’* [22]. This definition is coherent with current theories and describes habit as an explanatory mechanism to behaviour [23, 24]. The most traditional approach to habit formation involves repetition of a behaviour in a stable context [25] to the extent that, after sufficient repetition, the behaviour can be triggered by the cues in the environment rather than by having to make a conscious decision each time [26]. For example, a nurse might consciously decide to check patients’ feet for sensation and circulation during an annual diabetes review. After several repetitions of this examining behaviour, the behaviour becomes an automatic response to a cue (e.g. a pop-up prompt in the patients’ electronic record during a diabetes review). Furthermore, in the recent literature, a distinction has been made between habitual instigation (e.g. ‘choosing to provide weight management advice is something I do automatically’) and habitual execution (e.g. ‘once I have decided to provide weight management advice, giving weight management advice is something I do automatically’) [27]. Although, there is a level of variability in the way in which healthcare professionals deliver care, there are some behaviours that are performed repeatedly in a stable context, which may be to some extent habitual (e.g. examining feet).

Recently, Nilsen and colleagues [20] have called for research to explore strategies that could be used to help healthcare professionals with changing their habitual clinical behaviours (e.g. to replace old practices with new practices). Beyond the traditional repetition-based approaches to habit formation, two promising behaviour change techniques to create and break habit are action planning and coping planning [20, 28]. Experimental studies have shown that planning interventions can be used to facilitate habit formation by strengthening the association between contextual cues and goal-directed behaviours [29]. Action planning is a specific type of planning that has a scientific definition that differs from its lay usage. Action planning involves a person specifying very specifically when, where and how an intended behaviour will be performed. For example, ‘During annual reviews, I will use an educational leaflet to provide personalised nutrition advice to all patients with an above target body mass index (BMI)’ [30, 31]. Coping planning, i.e. problem solving, is sometimes used alongside action planning [32] and is another strategy that focuses on identifying potential barriers to an intended behaviour and (importantly) specifying how to overcome those barriers [30]. An example of a coping plan is ‘If the patient has difficulties reading the diabetes information leaflet, then I will ask a family member to read it out to the patient’. Research in clinical populations has shown that when used together, action and coping planning can be effective strategies for promoting various health behaviours including exercising and healthy eating [32, 33]. In healthcare professionals, one study tested the hypothesis that the relationship between healthcare professionals’ intention to provide guideline recommended care and self-reported clinical behaviour would operate indirectly through action and coping planning. The idea of a sequential reflective process underlying healthcare professional behaviour was confirmed for four of the six investigated behaviours [21]. In addition, the same study tested whether after accounting for that sequential process, an automatic process might operate in parallel. The automatic process was shown to operate alongside the sequential reflective process in four of six clinical behaviours [21].

Although there is evidence to suggest that healthcare professionals who make plans are more likely to enact clinical behaviours [21, 34], it is not clear through which mechanisms this change occurs. Action planning may function by making a specific cue more accessible in memory so that when the cue is encountered, healthcare professionals are more likely to remember and perform the behaviour [35]. For example, if healthcare professionals form a plan to provide self-management advice to patients with diabetes with high blood glucose levels, they will be more likely to recall and enact the behaviour automatically in ‘the heat of the moment’. When an action plan has been formed, the behaviour is more likely to be triggered automatically by the contextual cue (e.g. patient with high blood glucose levels) rather than by a slow, conscious contemplation process [36]. Coping planning may function similarly by linking a barrier with a solution (i.e. the barrier would serve as a cue that automatically triggers the solution to the barrier rather than disengagement from the behaviour altogether).

The present study is a secondary analysis drawing on data from the large i.e. the national ‘improving Quality in Diabetes’ (iQuaD) study data set [37]. The broader iQuaD study aimed to build a theoretical foundation to better understand the factors that underlie healthcare professional behaviour and to inform potential behaviour change interventions that target these factors [12, 21, 37]. The first analysis of the iQuaD data set aimed to test whether constructs from contemporary theories of behaviour (i.e. social cognitive theory, theory of planned behaviour, learning theory, action and coping planning) could predict healthcare professional behaviour [12]. The analysis found that theory-based constructs predicted multiple clinical behaviours in diabetes management. The second analysis further investigated whether the relationship between a reflective construct (i.e. intention) and healthcare professional behaviour operates indirectly through planning (action and coping planning) and whether habit operates in parallel alongside [21]. The findings showed that healthcare professionals who had higher intentions to perform recommended clinical behaviours were more likely to report enacting these behaviours in practice and that this relationship operated indirectly through planning (action and coping planning). Furthermore, the same analysis showed that both reflective (i.e. intention) and impulsive processes (i.e. habit) are predictive of multiple clinical behaviours [21]. Whilst the analysis supported a dual process conceptualisation of healthcare professional behaviour, the authors did not hypothesise how features of the reflective process (e.g. action and coping planning) may themselves serve to promote features of the impulsive process (e.g. habit); rather, the analyses focused on how habit operates alongside the reflective processes. Consistent with the broader literature on how action and coping planning (and implementation intentions) serve to create cue-response links to promote habit formation, the present study involved conducting a secondary analysis of iQuaD data to clarify the relationship between action/coping planning and habit in predicting healthcare professional behaviour. Although, previous analyses showed that planning (action and coping planning) is associated with healthcare professional behaviour [21], it remains unclear how this relationship operates. We hypothesised that the relationship between planning and clinical behaviour operates indirectly through habit. This hypothesis was tested across six guideline-recommended advising, prescribing and examining behaviours in the context of type 2 diabetes management in the UK primary care setting.

## Methods

### Design

A prospective correlational design was used to determine whether healthcare professionals performed six guideline-recommended clinical behaviours in the context of type 2 diabetes care. The study was a secondary analysis of the national ‘improving Quality in Diabetes’ (iQuaD) study dataset, which aimed to test theory-based determinants of healthcare professionals’ behaviour involved in managing type 2 diabetes in the UK primary care setting [13]. The six clinical behaviours selected for this study were (1) providing advice regarding weight management to patients with a BMI above 30 kg/m^2^, (2) prescribing additional antihypertensive drugs to patients whose blood pressure (BP) is 5 mmHg above 140 or 80 mmHg diastolic BP, (3) examining foot sensation and circulation, (4) providing advice about self-management, (5) prescribing additional therapy for glycaemic control in patients whose glycaemic haemoglobin A1c (HbA1c) is higher than 8% despite maximum dosage on two oral hypoglycaemic drugs, and (6) providing general education about diabetes. Following receipt of informed written consent, participating healthcare professionals were asked to complete self-reported measures of each theoretical construct at baseline and self-reported measures of the six guideline recommended practice behaviours at 12 months follow-up.

### Recruitment

As described in the published study protocol [38], practices were recruited through the UK Medical Research Council General Practice Research Framework (MRC GPRF). Initially, an invitation was sent to all GPRF practices in Scotland, Northern Ireland, Wales, and a random sample of practices in England, resulting in a total of 500 practices. Healthcare professionals from recruited practices were sent a written invitation to complete the baseline questionnaire. Respondents were then invited to complete self-reported measures of examining, prescribing and advising behaviours 12 months later.

### Survey administration

The baseline questionnaire included measures of various theoretical constructs [37]. To test the specific hypotheses in the present study, only measures of action planning, coping planning and habit for each of the six clinical behaviours were analysed. All measures of the theoretical constructs (e.g. action planning) were tailored specifically to each of the six behaviours (e.g. action planning for the clinical behaviour weight management advice: ‘I have a clear plan of how I will provide advice about weight management’). The questionnaire consisted of six sections each of which referred to a separate clinical behaviour. All relevant measures are summarised below, and the full baseline and follow-up questionnaire can be found in the additional files (see Additional files 1 and 2).

### Measures

A seven-point Likert scale ranging from *1-strongly disagree* to *7-strongly agree* was used to measure all theoretical constructs. Items forming each independent, mediating and dependent variable were developed and assessed separately for each of the six clinical behaviours. Higher scores represented cognitions in agreement with the behaviour. The development of the scale was directly based on the PRIME project, a theory-based study conducted with general medical and general dental practitioners [39]. The aim of PRIME was to apply well-established theories of behaviour to the experience of healthcare professionals, with the aim to identify modifiable variables that might be targets for intervention. This study examined the same theoretical constructs and used similar response formats; however, the item content was based on interviews and the behaviours were diabetes-focused.

*Habit* (*mediating variable*) was assessed with the four-item subscale of the Self-Reported Habit Index (SRHI; [40]): the Self-Reported Behavioural Automaticity Index (SRBAI; [41]). An example item utilising the scale is, ‘Providing advice about weight management to patients with a BMI above target is something I do automatically’. A higher score on the SRBAI indicates higher levels of habit/automaticity.

*Action planning* (*independent variable*) was measured using a previously validated three-item scale [30], modified to incorporate each of the clinical behaviours specified. An example of an action planning item utilised was ‘I have a clear plan of how I will provide advice about weight management’.

*Coping planning* (*independent variable*) was also measured with a previously validated 4 (i.e. for foot examination) to 12-item (i.e. for general education) scale [30]. Items were informed by a list of potential barriers to performing the six clinical behaviours. An example of a coping planning item utilised is ‘I have made a clear plan regarding providing advice about weight management to patients whose BMI is above target, if the clinic is busy and I am running 20 minutes late’.

All six *clinical behaviours* (*dependent variables*) were assessed at 12 months follow-up with six self-reported items, e.g. examining foot sensation and circulation: “Over the past 12 months, for approximately how many of the last 10 patients did you examine the circulation and sensation of their feet?” (See additional file 4 in [37] for all scale items).

### Analysis

**Fig. 1:**
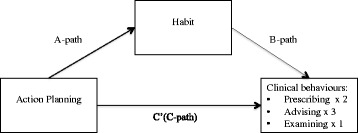
Indirect effect of action planning on clinical behaviours through habit. Path *a* is the direct effect of the predictor variable (action planning) on the mediator (habit). Path *b* is the direct effect of the mediator on the outcome variable (clinical behaviour). Path *c* is the direct effect of the predictor on the outcome variable. Path *c*’ is the indirect effect of the predictor variable on the outcome variable

**Fig. 2:**
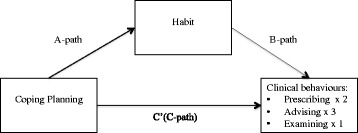
Indirect effect of coping planning on clinical behaviours through habit. Path *a* is the direct effect of the predictor variable (coping planning) on the mediator (habit). Path *b* is the direct effect of the mediator on the outcome variable (clinical behaviour). Path *c* is the direct effect of the predictor on the outcome variable. Path *c*’ is the indirect effect of the predictor variable on the outcome variable

We hypothesised that planning would exert its’ influence on healthcare professional behaviour through the psychological mechanism of habit. A mediation model was therefore used to test this hypothesis. In a mediation model, a variable *X* (planning) is assumed to be related to the outcome variable *Y* (healthcare professional behaviour), through the intervening variable called the mediator (habit) [42]. There are various methods that can be used to test mediation models including the *causal steps approach* [43] and the Sobel test [44]. An alternative to these approaches is the bootstrapping method [45], which involves repeatedly sampling from the data and estimating the indirect effect in each resampled data array. Simulation studies comparing different methods of mediation analysis have demonstrated bootstrapping to be superior to methods such as the Sobel test [44] or the causal steps approach [43], because it provides higher power whilst minimising type I error [46, 47]. We ran separate bootstrapped mediation analyses to test whether the relationship between action or coping planning and six clinical behaviours operated indirectly through their relationship with habit, resulting in 12 separate analyses (see Figs. 1 and 2). First bivariate correlations between all variables within each clinical behaviour were examined. Then a bootstrap method was used to test the significance levels of indirect effects for the hypothesised mediation models using Preacher and Hayes [48] INDIRECT macro. This is a computationally intensive procedure that involves repeatedly sampling from the data and estimating the indirect effect in each resampled data array. Simulation studies that assessed different methods of mediation analysis have found bootstrapping to be superior to methods such as the Sobel test [44] or the causal steps approach [43] because it provides higher power whilst minimising the incidence of type I error [46, 47]. Therefore, it was considered the most appropriate method to test the hypothesised mediation models. Since previous analyses of the same dataset found little evidence for clustering, it was decided that it would not be necessary to account for clustering in the current analysis [49].

## Results

### Response rates

The response rate for this study is reported at two levels, i.e. practice level and individual healthcare professional level [38]. At the practice level, one hundred practices (out of 500) consented and were recruited; one practice was subsequently excluded due to incomplete/unusable data. Thus, 99 practices consented and included healthcare professionals responding at baseline (19.8% practice-level response rate). At the healthcare professional level, 843 healthcare professionals from the 99 practices were invited to participate and 489 returned completed baseline questionnaires (326 GPs, 163 nurses) (58% healthcare professional level baseline response rate in the 99 recruited practices). Follow-up questionnaires were returned by 427 (289 GPs, 138 nurses) healthcare professionals (87% follow-up response).

### Descriptive statistics and correlations

Descriptive statistics can be found in Table 1. Ninety-nine percent of practice nurses and 45% of GPs were women. On average, GPs qualified in 1986 (SD = 8.50) and nurses in 1984 (SD = 8.25). Internal consistency measures for all measures are reported elsewhere [37]. Cronbach’s alpha for the construct measures ranged from 0.70 to 0.97. Although healthcare professionals reported performing each behaviour with the majority of their patients, there was considerable variability between healthcare professionals within and across behaviours. The scale mid-point of all the theoretical constructs was exceeded, showing a tendency of favouring the behaviour [37]. Table 1 shows bivariate associations between all variables within all six behaviours. The size of the associations between the predictor variables (action and coping planning) and the mediator (habit) was medium (large for foot examination), and associations between the variables within each process were medium to large.

**Table 1:** Descriptive statistics and correlations between theoretical predictors and self-reported behaviours

Providing advice regarding weight management to BMI above a target of 30 kg/m^2^ (*N* = 424)				
	1	2	3	4
1. Behaviour	7.80 (2.48)			
2. Action planning	0.14**	5.88 (0.92)		
3. Coping planning	0.28**	0.31**	4.45 (1.26)	
4. Habit	0.37**	0.27**	0.49**	4.81 (1.29)
Prescribing to reduce blood pressure to 140/80 mm Hg (*N* = 335)				
	1	2	3	4
1. Behaviour	6.34 (2.64)			
2. Action planning	0.37**	5.91 (0.84)		
3. Coping planning	0.46**	0.48**	4.61 (1.22)	
4. Habit	0.51**	0.31**	0.49**	3.97 (1.33)
Examining the feet (*N* = 218)				
	1	2	3	4
1. Behaviour	6.96 (3.45)			
2. Action planning	0.37**	6.22 (0.99)		
3. Coping planning	0.46**	0.64**	5.53 (1.49)	
4. Habit	0.71**	0.41**	0.53**	4.36 (1.73)
Providing diabetes self-management advice (*N* = 332)				
	1	2	3	4
1. Behaviour	7.69 (2.58)			
2. Action planning	0.29**	5.44 (1.16)		
3. Coping planning	0.37**	0.61**	4.71 (1.36)	
4. Habit	0.37**	0.51**	0.58**	4.87 (1.51)
Prescribing to reduce HbA1c levels to <8.0% (*N* = 288)				
	1	2	3	4
1. Behaviour	6.88 (2.71)			
2. Action planning	0.26**	5.62 (1.08)		
3. Coping planning	0.26**	0.67**	4.76 (1.31)	
4. Habit	0.29**	0.41**	0.51**	4.01 (1.46)
Providing diabetes-related education (*N* = 346)				
	1	2	3	4
1. Behaviour	7.76 (2.61)			
2. Action planning	0.43**	5.58 (1.17)		
3. Coping planning	0.34**	0.64**	4.49 (1.26)	
4. Habit	0.33*	0.55*	0.56**	4.91 (1.50)

Table was adapted from [12]. Permission from the authors has been obtained

Means (SD) presented along the diagonal

*p < 0.05; **p < 0.01

### Model testing

We hypothesised that there would be an indirect effect of action planning and coping planning on each of the six guideline-recommended behaviours in type 2 diabetes care through habit (the mediator variable). In 12 separate analyses, the 95% confidence intervals of the indirect effects were obtained with 5000 bootstrap resamples [48]. All planning-behaviour relationships were shown to operate through habit. The estimates for the direct and indirect effects are shown in Table 2. In six out of the 12 analyses, the relationships between planning and behaviour were no longer significant when the indirect effect via habit was accounted, indicating a full mediation effect.

**Table 2:** Bootstrap analysis of the magnitude and statistical significance of the direct and indirect effects

Independent variable	Mediator variable	Dependent variable	B unstandardised a-path	B unstandardised b-path	B standardised indirect effect	SE	95% CI (lower, upper)
AP	Habit	Weight management advice	0.37***	0.62***	.23	0.05	0.15, 0.34
CP	Habit	Weight management advice	0.49***	0.57***	.28	0.05	0.20, 0.38
AP	Habit	Prescribing additional antihypertensive drug	0.43***	0.47***	.21	0.06	0.10, 0.34
CP	Habit	Prescribing additional antihypertensive drug	0.54***	0.51***	.28	0.07	0.14, 0.43
AP	Habit	Examining feet	0.84***	1.04***	.88	0.15	0.61, 1.22
CP	Habit	Examining feet	0.68***	0.93***	.63	0.09	0.47, 0.83
AP	Habit	Advise about self-management	0.65***	0.45***	.29	0.07	0.16, 0.45
CP	Habit	Advise about self-management	0.62***	0.36***	0.23	0.06	0.11, 0.36
AP	Habit	Prescribe HbA1c	0.58***	0.34***	.20	0.06	0.09, 0.34
CP	Habit	Prescribe HbA1c	0.58***	0.33***	.19	0.06	0.14, 0.45
AP	Habit	Provide general education	0.67***	0.23**	.15	0.06	0.05, 0.27
CP	Habit	Provide general education	0.64***	0.32***	0.20	0.06	0.09, 0.32

As none of the 95% confidence intervals for the estimate of indirect effects included zero, there is a statistically significant indirect effect of action planning and coping planning on all six clinical behaviours through habit

*AP* action planning, *CP* coping planning

**p < 0.01; ***p < 0.001

SE = standard error

## Discussion

The aim of the current study was to investigate whether the relationship between action planning and coping planning and six guideline-recommended clinical behaviours in the context of type 2 diabetes care is mediated by habit. As hypothesised, healthcare professionals who scored higher on planning (action or coping plan) for providing advice, prescribing or examining feet were more likely to report performing such care (consistent with previous analyses) and this relationship operated indirectly through habit, which to our knowledge is the first time this has been demonstrated in healthcare professional populations and across multiple behaviours form the same population. This paper directly addresses calls from the literature for empirical tests of how habit relates to healthcare professional behaviour [20]. Specifically, this study shows that habit and planning are two important constructs to consider when targeting change in healthcare professional behaviour, and the mechanism by which planning may have its effect on behaviour is through habit.

These findings add to two previous analyses of the iQuaD data set [12, 21, 37]. The first analysis showed that theory-based constructs can predict multiple clinical behaviours in the context of diabetes management [12]. The second analysis showed that healthcare professionals who are more motivated to enact recommended clinical behaviours are more likely to report performing those behaviours and that the mechanism underlying this relationship is planning (action and coping planning). Furthermore, this second analysis supported the idea of a reflective-impulsive process, represented by habit and intention, which underlies healthcare professional behaviour. One question that resulted from these first two analyses was how, or through what mechanism, planning (coping and action planning) relates to clinical behaviour. The current analysis provides the first evidence that the mechanism underlying the positive association between planning (action and coping planning) and clinical behaviour is habit. Given the correlational design of the study, it is not possible to make any causal inferences about the direction of the relationship between planning and habit; however, our findings provide useful theoretical insights with implications for healthcare professional behaviour change.

The positive relationship identified between action planning and clinical behaviour and this operating indirectly through habit is consistent with the literature on implementation intentions (i.e. specific ‘if-then’ plans) [36]. It may be that healthcare professionals who form an action plan through a process of conscious deliberation create a mental link between a cue in the clinical context and a goal-directed behaviour. Once the cue is encountered (e.g. during the consultation), the healthcare professional may be more likely to perform the planned behaviour as an automatic response to that cue. We also found that healthcare professionals who scored higher on coping planning were more likely to report executing guideline recommended clinical behaviours even when faced with barriers. Again, the positive relationship between coping planning and clinical behaviour operated indirectly through the mechanism of habit. It is probable that the mechanism underlying coping planning is comparable to action planning in that a mental link is formed between a (risk) situation and an appropriate behavioural response (coping plan). Furthermore, coping planning might promote habit formation indirectly by supporting behavioural maintenance in the face of potential obstacles [32]. Both the linkage of a risk situation with an appropriate coping response and maintained behavioural performance could contribute to the process of habit formation in the clinical context.

There are several reasons why it may be useful to promote habit formation in healthcare professionals in the primary care setting. Healthcare professionals have limited time available during consultations and often have to make numerous skilled decisions. Once a behaviour has become habitual, it can proceed quickly and efficiently in response to contextual cues [50, 51] rather than having to rely on slow, more cognitively demanding processes. For example, one guideline recommended practice in diabetes care involves prescribing medication to reduce blood pressure. The initiation of this behaviour is often preceded by an explicit cue (i.e. blood pressure target not met) and could therefore be elicited habitually. Once the behaviour has been initiated, more deliberative decision-making can be utilised to decide/agree on the specific medication regime. This example is in line with dual process models which suggest that behaviour is driven by both reflective and impulsive processes which operate in parallel [50]. Furthermore, habit is useful as a behavioural determinant to healthcare professional behaviour [20]. The dominant theories used to predict healthcare professional behaviours focus on concepts that are part of the reflective pathways to behaviour (e.g. attitudes, norms, intention and self-efficacy). By focusing on the reflective pathway only, there is a risk of neglecting important aspects of the variance of healthcare professional behaviour, a proportion of which can be explained by impulsive processes such as habit.

### Strengths and limitations

We tested our mediation models across six different guideline-recommended behaviours in type 2 diabetes care. To test the mediation models, we used state-of-the-art bootstrapped mediation analysis, which is superior to traditional methods of mediation analysis and therefore is considered a strength of this research [43, 44, 46]. Bootstrapping is based on an estimate of the indirect effect; however, compared to the Sobel test, it makes no assumptions about the sampling distribution of the indirect effect, making it a more flexible approach [42]. For bootstrapping, no standard error is needed to make the inference, bypassing the problem of how to optimally estimate the standard error of the indirect effect [42]. All theoretical measures had the same level of specificity using the TACT (Target, Action, Context, and Timing) principle and corresponded with the clinical behaviours. Furthermore, although previous research has shown that planning plays a post-intentional role and can promote the enactment of recommended clinical behaviours [21, 34], this is the first study to show that habit may be the mechanism underlying the relationship between planning and clinical behaviour. Given the consistency of this result across both planning cognitions and six guideline-recommended behaviours, one would expect that these results could translate to other clinical behaviours across different healthcare sectors (e.g. secondary and tertiary care). A limitation of this study involved the cross-sectional assessment of planning and habit. Cole and Maxwell have called this a half-longitudinal design and emphasise that this might introduce a source of bias to the observed effect [48, 49]. Furthermore, the observational nature of the study and the fact that planning (action and coping planning) and habit were both measured at the same time does not allow us to draw any causal inferences about the direction of the relationships. Future research could explore this mediation model in a longitudinal design where all variables (independent, mediator and dependent variable) are measured at different time points or else alongside a randomised trial design which would allow for a more robust assessment of the causal mechanisms underlying planning. A further limitation of this research was that habit and healthcare professional behaviour were measured through self-report. Measuring habit through self-report assumes that individuals can be aware of the degree of habit strength of a given behaviour by reflecting on the consequences of their actions [23, 52]. Despite this limitation, the Self-Reported Behavioural Automaticity Scale has shown to be a reliable measure that is consistent with recent theoretical definitions of habit [24]. Future studies could explore qualitative research methods to observe habitual behaviours in the clinical context. Video observations and conversation analysis might offer a promising way to assess cues and automatic behaviours by studying interactions, paying attention to both verbal and non-verbal cues [53]. This is a data-driven process through which habitual patterns of interaction can be identified; therefore, it could be useful for observing and changing habitual behaviours in clinical practice through feedback provision. Measuring behaviour through self-report is another potential source of bias, and it cannot be ruled out that healthcare professionals over-reported the extent to which they had delivered a specific aspect of care. This study focused on the behaviour of individual healthcare professionals, yet healthcare is often delivered by teams/groups. Therefore, it would be beneficial to test our model using different ways of aggregating the individual habit scores. For example, a multilevel modelling approach could be used to account for both individual and practice-level clustering of habit [49]. The individual baseline response rate of 58% is higher than what was achieved in previous theory-based questionnaire surveys [54], possibly due to the recruitment of practices that may be more motivated (which may have reduced the representativeness) and the use of remuneration for time spent completing the questionnaire.

### Implications for intervention design

From a behavioural perspective, the issue of implementation can be conceptualised as a need to create new clinical routines or habitual behaviours. Similarly, de-implementation can be conceptualised as the need for ‘breaking’ old routines. Our findings offer some suggestions that might be useful for developing behaviour change interventions that are in line with practice guidelines and ‘breaking’ outdated routines. This research shows that action and coping planning may support clinical behaviour by creating cue-response links that underlie habit [34]. There are various modes through which an action and coping planning intervention could be used to support healthcare professionals with changing their routines. Interventions could be delivered with the help of planning sheets that include pre-specified situations and solutions or could be self-formulated [55]. Although independent planning is easier and more cost effective, monitored and supervised planning (e.g. using telephone assistance) allow for controlling the quality of the plans, which is essential for effective behaviour change [55]. In cases where monitoring is not possible, the use of planning help sheets could be another intervention option. These planning sheets could include pre-specified opportunities to enact recommended clinical behaviours and ways in which these behaviours could be performed in the clinical context. Similarly, a planning sheet could include barriers to good practice and possible ways of coping with these barriers. Furthermore, qualitative research methods (e.g. interviews or video observations) could be used to identify both contextual cues and/or barriers to good practice that could be used to inform a planning sheet, minimising the demands on healthcare professionals, whilst maximising the quality of potential plans.

## Conclusions

To our knowledge, this is the first study that has tested the role of habit as a mediator of the planning-behaviour relationship in a large sample of healthcare professionals. We have found that the relationship between planning and six guideline-recommended prescribing, examining and advising behaviours operated indirectly through habit. Given the challenges of implementing guideline recommended care and de-implementing outdated care within time constrained practice environments, our findings have the potential to inform the development of novel interventions that target habit to promote improved healthcare.

## Additional files

Additional file 1: Baseline postal questionnaire incorporating organisational and clinical questionnaires and behaviour simulation measures. (PDF 827 kb)
Additional file 2: Twelve-month clinician self report behaviour questionnaire. (PDF 75 kb)

## Abbreviations

BMI: Mody mass index
GP: General practitioner
iQuaD: Improving Quality in Diabetes study
PRIME: Process modelling in implementation research
SRBAI: Self-reported behavioural automaticity scale
SRHI: Self-reported habit index
TACT: Target, action, context, timing
TPB: Theory of planned behaviour
UK: United Kingdom
